# Evaluation of pre-mir-34a rs72631823 single nucleotide polymorphism in triple negative breast cancer: A case-control study

**DOI:** 10.18632/oncotarget.26385

**Published:** 2018-12-11

**Authors:** Despoina Kalapanida, Flora Zagouri, Maria Gazouli, Eleni Zografos, Constantine Dimitrakakis, Spyridon Marinopoulos, Aris Giannos, Theodoros N. Sergentanis, Efstathios Kastritis, Evangelos Terpos, Meletios-Athanasios Dimopoulos

**Affiliations:** ^1^ Department of Clinical Therapeutics, Alexandra Hospital, Medical School, University of Athens, Athens, Greece; 2 Department of Basic Medical Sciences, Laboratory of Biology, University of Athens School of Medicine, Athens, Greece; 3 Laboratory of Cell and Gene Therapy, Biomedical Research Foundation of the Academy of Athens, Athens, Greece; 4 Department of Obstetrics and Gynaecology, Alexandra Hospital, Medical school, University of Athens, Athens, Greece

**Keywords:** SNPs, pre-miR-34a, miRNA, triple negative breast cancer, biomarker

## Abstract

**Aim:**

The purpose of this study is to evaluate the role of pre-miR34a rs72631823 as potential risk factor and/or prognostic marker in patients with triple negative breast cancer.

**Methods:**

114 samples of DNA from paraffin embedded breast normal tissues of patients with triple negative breast cancer and 124 samples of healthy controls were collected and analyzed for pre-miR34a rs72631823 polymorphism.

**Results:**

Pre-miR34a rs72631823 A allele was associated with increased TNBC risk both in univariate and multivariate analysis. The number of pre-miR34a rs72631823 AA subjects was very small and the association did not reach significance (*p* = 0.176, Fisher’s exact test). The examined polymorphism was not associated with overall survival at the univariate or multivariate Cox regression analysis (adjusted HR = 1.60, 95%CI: 0.64–3.96 for miR34 rs72631823 GA/AA vs. GG).

**Conclusion:**

Our case-control study suggests that pre-miR34a rs72631823 A allele is associated with increased triple negative breast cancer risk.

## INTRODUCTION

Triple negative breast cancer (TNBC) represents 15–20% of all invasive breast cancers and is characterized by high biological heterogeneity with increased levels of distal recurrence and poor survival, besides high response rate to chemotherapy that constitutes the only standard of treatment [1]. The discovery of new biomarkers related to TNBC can lead to a better understanding of the disease and to the design of new targeted therapies with the aim to improve the outcome of this malignancy [2, 3]. Single nucleotide polymorphisms (SNPs) have been implicated in cancer development, prognosis and drug-resistance, often interacting with other factors [4]. Regarding TNBC, several SNPs in extensively studied genes, such as BRCA1 [5], PARP1 [6], ERCC2 [7], TNFa [8], TMPRSS6 [9], GLCE [10], have been associated with the disease across different geographic regions and races.

MicroRNAs are small, endogenous, non-coding RNAs of approximately 22 nucleotides that could regulate gene expression at post-transcriptional level by binding with mRNAs. Depending on the complementarity between the seed sequence of a microRNA (region of 6-8 nucleotides in the 5’ end of microRNA) and mainly the 3’untranslated region of the target mRNA molecule, microRNAs could induce degradation or translational repression of their targets [11, 12]. Through this action, microRNAs play a pivotal role in key cellular processes, such as proliferation, differentiation, epithelial-mesenchymal-transition, embryogenesis, angiogenesis, invasion and apoptosis [13, 14]. MicroRNAs were first described in the literature by Lee et al [15], and increasing evidence has shown that expression of microRNAs is deregulated in multiple cancers (lung, ovarian, colorectal, breast cancer etc.) [14, 16–18]. Depending on their target, microRNAs may function as oncogenes or tumor suppressor genes [19], and are associated with cancer development, progression, metastatic index and drug resistance [17, 20].

Sequence alterations in pre-miRNA molecules seem to affect the levels of the mature microRNA [21]. Pre-miR-34a rs72631823 polymorphism is observed in the terminal loop of the pre-miR-34a. The presence of the A allele (rather than the G allele) correlates with increased pre-miR-34 sensitivity to processing by Drosha and Dicer, resulting in increased levels of miR34a in the cytoplasm. This observation was reported by Locke JM et al [22] in a cell line of pancreatic beta cells, where the presence of pre-miR-34a rs72631823 A allele was associated with increased levels of miR-34a.

This study was designed in order to investigate the role of pre-miR-34a rs72631823 polymorphism as a potential risk factor and/or a prognostic marker in patients with TNBC who have received chemotherapy, via a case-control study of 114 patients with triple negative breast cancer and 124 controls.

## RESULTS

Table 1 presents descriptive statistics regarding demographic lifestyle and reproductive parameters, in cases and controls. Cases were younger at menarche (*p* = 0.023, MWW) and consumed alcohol more frequently (*p* = 0.046, Chi-square test), compared to controls. No significant differences were documented in education, menopausal status, smoking rates between cases and controls. The majority of TNBC cases were T2 (61.4%), node-negative (63.2%), grade 3 (86.9%) carcinomas.

**Table 1:** Distribution of the 114 TNBC cases and the 124 age-matched controls by demographic, lifestyle and reproductive variables

Variable	Cases	Controls	
Continuous variables	Mean (SD)	Mean (SD)	*p*-value
Age (years)	56.1 (14.3)	56.6 (13.9)	matched variable
Age at menarche (years)	12.9 (1.8)	13.4 (1.6)	0.023^MWW^
Categorical and ordinal variables	**N (%)**	**N (%)**	
Education			0.103^C^
*Uneducated / Primary*	10 (8.8)	17 (13.7)	
*Secondary*	14 (12.3)	27 (21.8)	
*High School*	59 (51.7)	54 (43.6)	
*College / University*	31 (27.2)	26 (21.0)	
Menopausal status			0.404^C^
*Premenopausal*	34 (29.8)	31 (25.0)	
*Postmenopausal*	80 (70.2)	93 (75.0)	
Ever smoking			0.399^C^
*Yes*	36 (31.6)	33 (26.6)	
*No*	78 (68.4)	91 (73.4)	
Alcohol consumption			0.046^C^
*< 1 glasses/week*	75 (65.8)	96 (77.4)	
*≥ 1 glasses/week*	39 (34.2)	28 (22.6)	
Tumor size		
*T1*	32 (28.1)		
*T2*	70 (61.4)		
*T3*	8 (7.0)		
*T4*	4 (3.5)		
Nodal status			
*N0*	72 (63.2)		
*N1*	13 (11.4)		
*N2*	9 (7.9)		
*N3*	20 (17.5)		
Grade			
*G1*	3 (2.6)		
*G2*	12 (10.5)		
*G3*	99 (86.9)		
Histology			
*Ductal*	87 (76.3)		
*Lobular*	10 (8.8)		
*Other*	17 (14.9)		

MWW: *p*-value derived from Mann-Whitney-Wilcoxon test for independent samples; C: *p*-value derived from Chi-square test

Genotype frequencies, unadjusted and adjusted ORs for the examined polymorphisms are provided in Table 2. At the univariate analysis, Pre-miR34a rs72631823 A allele was associated with increased TNBC risk (crude OR = 3.13, 95%CI: 1.67–5.87 in the allele dose-response model and crude OR = 2.98, 95%CI: 1.56–5.70 for the GA vs. AA comparison). The number of pre-miR34a rs72631823 AA subjects was very small and the association did not reach significance (*p* = 0.176, Fisher’s exact test). The multivariate analysis, adjusting for age, smoking, alcohol consumption, menopausal status, age at menarche and education, confirmed that Pre-miR34a rs72631823 A allele was associated with increased TNBC risk (adjusted OR = 2.89, 95%CI: 1.53–5.47 in the allele dose-response model; adjusted OR = 2.56, 95%CI: 1.30–5.03 for the GA vs. AA comparison).

**Table 2:** Genotype frequencies and odds ratios regarding the association between Pre-miR34 rs72631823 polymorphism and TNBC risk

Genotype	Cases	Controls	OR (95% CI)^a^	OR (95% CI)^b^
	**N (%)**	**N (%)**		
**Total study**				
*GG*	76 (66.7)	107 (86.3)	1.00 (Ref.)	1.00 (Ref.)
*GA*	36 (31.6)	17 (13.7)	**2.98 (1.56–5.70)**	**2.56 (1.30–5.03)**
*AA*	2 (1.7)	0 (0.0)	Not estimable due to zero controls^§^	Not estimable due to zero controls
*Allele dose-response*			**3.13 (1.67–5.87)**	**2.89 (1.53–5.47)**
**Premenopausal women**			**OR (95% CI)**^a^	**OR (95% CI)**^c^
*GG*	22 (64.7)	28 (90.3)	1.00 (Ref.)	1.00 (Ref.)
*GA*	11 (32.4)	3 (9.7)	**4.67 (1.16–18.80)**	4.03 (0.83-19.52)
*AA*	1 (2.9)	0 (0.0)	Not estimable due to zero controls	Not estimable due to zero controls
*Allele dose-response*			**4.89 (1.27–18.93)**	**5.15 (1.22–21.68)**
**Postmenopausal women**			**OR (95% CI)**^a^	**OR (95% CI)**^c^
*GG*	54 (67.5)	79 (85.0)	1.00 (Ref.)	1.00 (Ref.)
*GA*	25 (31.3)	14 (15.0)	**2.61 (1.25–5.48)**	**2.27 (1.06–4.88)**
*AA*	1 (1.2)	0 (0.0)	Not estimable due to zero controls	Not estimable due to zero controls
*Allele dose-response*			**2.73 (1.33–5.60)**	**2.49 (1.20–5.16)**

§*p* = 0.176 for the association, Fisher’s exact test; a: unadjusted OR; b: OR adjusted for age, smoking, alcohol consumption, menopausal status, age at menarche and education; c: OR adjusted for age, smoking, alcohol consumption, age at menarche and education.

Bold cells denote statistically significant associations.

Subgroup analyses by menopausal status reproduced the findings of the overall analysis. In premenopausal women, the adjusted OR for the allele dose-response model was 5.15 (95%CI: 1.22–21.68). Accordingly, in postmenopausal women the adjusted OR for the allele dose-response model was 2.49 (95%CI: 1.20–5.16).

No significant deviation from HWE was documented for the examined polymorphism (Pearson’s chi2(1) = 0.67, *p* = 0.413).

**Figure 1:**
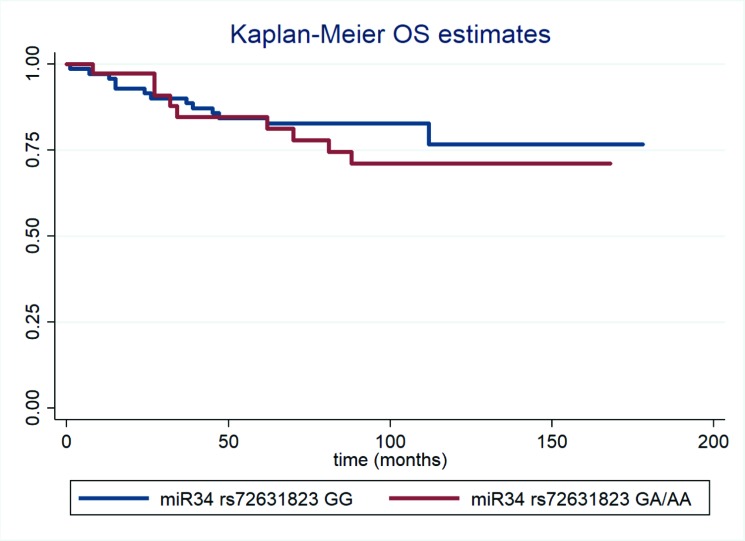
Kaplan–Meier overall survival estimates for Pre-miR-34 rs72631823 GG (blue lines) and GA/AA TNBC cases.

The results of the nested prospective study in cases are shown in Table 3. The median follow-up was equal to 9.3 years; the examined polymorphism was not associated with overall survival at the univariate or multivariate Cox regression analysis (adjusted HR = 1.60, 95%CI: 0.64–3.96 for miR34 rs72631823 GA/AA vs. GG; Table 3). Figure 1 presents Kaplan–Meier overall survival curves for the studied polymorphism.

**Table 3:** Results of the univariate and multivariate Cox regression analysis examining the associations between Pre-miR34 rs72631823 polymorphism and overall survival in women with TNBC

Genotype	Cases	Univariate HR (95% CI)	Multivariate HR (95% CI)^§^
	N (%)		
miR34 rs72631823			
*GG*	76 (66.7)	1.00 (Ref.)	1.00 (Ref.)
*GA/AA*	38 (33.3)	1.28 (0.55–2.96)	1.60 (0.64–3.96)

§adjusted for age, grade and stage

## DISCUSSION

This study is the first to highlight that pre-miR34a rs72631823 A allele is associated with nearly 3-fold increased risk of TNBC. The association was evident in premenopausal as well as postmenopausal women and persisted after adjustment for various potential confounders, including age, smoking, alcohol consumption, age at menarche and education. On the other hand, pre-miR34a rs72631823 A allele did not seem to alter the overall survival of TNBC.

This is the first study that evaluates the role of pre-mir34a rs72631823 polymorphism as a potential risk factor or/and prognostic factor in TNBC. Since the examined polymorphism has been previously investigated only once in a line of pancreatic beta cells, and not in cancer, based on current knowledge our results cannot be compared to other studies. However, these findings seem to agree with previous studies stating that alterations in pre-miRNAs could affect the expression levels of genes involved in oncogenesis. The association trend between pre-mir34a rs72631823 and TNBC is in accordance with the studies of Morales S et al [25] and Li M et al [26] that present the association of single nucleotide polymorphisms in pre-miRNAs with breast cancer in a South American population and gastric cancer in a Chinese population. Pre-miRNA polymorphisms seem to affect oncogenesis by modifying the cellular levels of mature miRNA, as it is mentioned in the study of Lv H and his colleges [21].

Since 2005, when Iorio et al [16] presented the first miRNA signature in breast cancer, several miRNAs including miR-200, miR-21, miR-34, miR-31, miR-146 have been implicated in important cellular processes such as migration, proliferation, EMT and apoptosis both in luminal and basal phenotype [27–30]. MicroRNAs could serve as excellent biomarkers, since they have great stability in tissues and human fluid and they could be easily detected in tumor biopsies [21, 31–34]. MiRNA 34 constitutes of a highly conserved family of 3 miRNAs through evolution; miRNA34a that is encoding from a transcriptional unit on chromosome 1p36 and miRNAs34b/c that are encoding from the same transcriptional unit on chromosome 11q23 [35]. MiRNA34a, the most extensively studied miRNA of this family, is a tumor suppressor as it has been found to be down-regulated in multiple tumors [36, 37]. In 2007, several studies indicated the role of mir-34a in inducing apoptosis and cell-cycle arrest though its regulation of the p53 protein [35, 38–42]. In the next few years, more studies established mir-34a’s association with key processes in carcinogenesis such us inhibition of proliferation, colony formation, migration and drug resistance, as well as the enhancement of cell-cycle arrest, senescence and apoptosis [36, 43] in hematologic malignancies [44], lung cancer [45], brain tumor [46], HHC [47, 48], ovarian cancer [49] and in other solid tumors [50–52]. Regarding breast cancer, mir-34a has already been proven to regulate cell proliferation, differentiation, epithelial-mesenchymal-transition, apoptosis, cell cycle arrest and to reverse drug resistance, after interaction with significant signaling pathways such as MET, p53, NOTCH, TGF-β, PRKD1 and BCL-2 pathways [53–56]. Furthermore, there are indications that mir-34a is associated with histologic grade, while there has been no correlation with clinicopathology features in breast cancer up until now [57, 58].

An important element of validity of the present study is the absence of deviation of allele frequencies in controls for pre-mir34a rs72631823 concerning HWE. Discrepancies on HWE might indicate incorrect or guided sample selection by the research team, genotyping errors or incongruity of mating. Despite the originality, limitations of this case-control study should be reported. First, more studies with increased sample size should be performed to overcome the obstacle of the small number of homozygous pre-mir34a rs72631823 AA subjects. Additionally, in our study, the investigated polymorphism was evaluated in paraffin embedded normal breast tissue, which however is a very common source for DNA extraction in the studies regarding microRNAs.

In conclusion, according to our data it seems that pre-mir34a rs72631823 A allele is associated with increased TNBC risk. Future well designed studies should be performed to elucidate the mechanisms underlying the effect of this SNP in TNBC, across different races.

## MATERIALS AND METHODS

### Subjects

Paraffin embedded breast normal tissues were collected from 114 patients with histologically confirmed TNBC during the period 2000-2014. Operations were conducted at the Gynecology Department, “Alexandra” Hospital, Medical School, University of Athens, Greece and patients received chemotherapy at the Oncology Department, “Alexandra” Hospital, Medical School, University of Athens, Greece. The exclusion criteria were as follows: metastatic disease at diagnosis, no invasive disease, family history of breast cancer (1st degree relative with breast cancer, known BRCA1 and BRCA2 mutations), history of prior malignancy and no signed informed consent form. Additional information regarding the histology, tumor size, grade, histological stage, lymph node infiltration, the expression levels of ki67 and p53, disease free survival (DFS) and overall survival (OS) were registered in an electronic database according to the patients’ medical records. Concerning controls, women with negative results for breast cancer on routine mammography test were selected. Controls were matched on age (+– 2 years) with patients and had no history of other malignancy. Cases and controls were Caucasian and lived in the same geographical region (greater metropolitan area of Athens, Attica). Informed consent was obtained from all individual participants included in the study. This case-control study has been approved by the local Institutional Review Board.

### Genotyping of pre-mir-34a rs72631823

DNA from paraffin embedded normal breast tissues of patients and DNA from the blood of healthy controls was isolated using the Nucleopsin Tissue kit (Macherey Nigel, Germany). Amplification of the selected sequences was performed with allele-specific PCR (polymerase chain reaction), using two allele-specific reverse primers: RG, 5′ CTTGCTGATTGCTTCCTTAC 3′ for the wild type allele and RA: 5′ CTTGCTGATTGCTTCCTTAT 3′ for the mutant allele, in combination with a common forward primer F: 5′ CACATTTCCTTCTTATCAACAG 3′ in two separate PCR reactions. The 3′-ends of the reverse primers were able to anneal to the regions that differed between the two alleles. The endogenous levels of the corresponding gene products were quantified by ELISA.

### Statistical analysis

Data were cross-tabulated by case-control status; Mann-Whitney Wilcoxon test (MWW) and Chi-square test were used for the comparison of demographic, lifestyle and reproductive factors in cases and controls, as appropriate. To analyze the associations between the examined polymorphisms and the risk of TNBC, three separate logistic regression models were evaluated: heterozygous vs. wild type (the most frequent homozygous genotype was set as “wild type”), homozygous vs. wild type and dose-response allele model (0: wild type, 1: heterozygous, 2: homozygous subjects). Unconditional logistic regression analysis was performed to estimate univariate and multivariate odds ratios (ORs) with 95% confidence intervals (CIs). The multivariate ORs were adjusted for age, smoking, alcohol, menopausal status age at menarche and education. In addition, subgroup analyses for premenopausal and postmenopausal women were conducted. The deviation of allele frequencies in controls from the Hardy-Weinberg Equilibrium (HWE) was examined with the appropriate goodness-of-fit Chi-square test, given that the deviation may denote bias [23]. Univariate and multivariate Cox regression analysis was performed to evaluate the association of polymorphisms with overall survival in breast cancer patients; the multivariate Cox regression model was adjusted for patient age, grade (increment by one in the low=1, intermediate=2, high=3 grouping) and stage (increment by one in the I-II-III TNM classification) of breast cancer [24]. Kaplan–Meier survival curves were estimated to graphically represent the results. Censoring date was January 31, 2016. Statistical analysis was performed using STATA/SE version 13 statistical software (Stata Corporation, College Station, TX, USA).
